# Epigenetic silencing of TMEM176A promotes esophageal squamous cell cancer development

**DOI:** 10.18632/oncotarget.19550

**Published:** 2017-07-25

**Authors:** Ying Wang, You Zhang, James G. Herman, Enqiang Linghu, Mingzhou Guo

**Affiliations:** ^1^ Department of Gastroenterology & Hepatology, Chinese PLA General Hospital, Beijing 100853, China; ^2^ Department of Gastroenterology, The Affiliated Fu Xing Hospital of Capital Medical University, Beijing 100038, China; ^3^ The Hillman Cancer Center, University of Pittsburgh Cancer Institute, Pittsburgh, PA 15213, USA

**Keywords:** TMEM176A, esophageal squamous cell cancer, DNA methylation, epigenetics, tumor suppressor

## Abstract

The function of human transmembrane protein 176A (TMEM176A) in cancer remains unclear. To understand the function and mechanism of TMEM176A in human esophageal cancer development, 13 esophageal cancer cell lines and 267 cases of primary esophageal squamous cell cancer (ESCC) samples were analyzed by methylation specific PCR (MSP), flow cytometry, immunohistochemistry and transfection assays. TMEM176A was highly expressed in BIC1 cells and loss of TMEM176A expression was found in TE1, TE3, TE13, KYSE140, KYSE180, KYSE410, KYSE450, KYSE520, Segl, KYSE150, YES2 and COLO680N cells. Complete methylation was detected in TE1, TE3, TE13, KYSE140, KYSE180, KYSE410, KYSE450, KYSE520, Segl, KYSE150, YES2 and COLO680N cells, while unmethylation was detected in BIC1 cells. Restoration of TMEM176A expression was induced by 5-aza-2’-deoxycytidine treatment in methylated cell lines. TMEM176A was methylated in 66.7% (178/267) of primary esophageal cancer samples, and promoter region methylation was significantly associated with tumor differentiation (*p*<0.001) and loss off/reduced expression of TMEM176A (*p*<0.05). Methylation of TMEM176A was significantly associated with poor 5-year overall survival (*p* < 0.05). Cox proportional hazards model analysis suggest that TMEM176A methylation is an independent prognostic factor for poor 5-years OS. TMEM176A inhibited cell invasion and migration, and induced apoptosis in esophageal cancer cells. TMEM176A suppressed esophageal cancer cell growth both *in vitro* and *in vivo*. In conclusion, TMEM176A is frequently methylated in human ESCC and the expression of TMEM176A is regulated by promoter region methylation. TMEM176A methylation may serve as a diagnostic and prognostic marker in ESCC. TMEM176A is a potential tumor suppressor in human ESCC.

## INTRODUCTION

Esophageal cancer (EC) is one of the most common malignancies. The overall 5-year survival ranges from 15% to 25% [1]. Esophageal squamous cell cancer (ESCC) is the major tissue type of EC and accounts for 90% of cases worldwide [2]. The so-called “Asian Esophageal Cancer Belt” encompasses areas including Turkey, Iran, Kazakhstan and northern and central China, with an estimated incidence of more than 100 cases/100,000 people per year [3–5]. Smoking, alcohol and nitrates are regarded as major risk factors of esophageal cancer [6]. Environmental factors may play important roles in esophageal carcinogenesis [7–9]. Beyond lifestyle, the role of environmental chemicals as determinants of DNA methylation has gained considerable attention [10–13]. Epigenetics may play more important roles than genetics in esophageal cancer. For example, CHFR, a DNA damage repair gene, is frequently methylated but rarely mutated in human ESCC [14, 15].

Human transmembrane protein 176A (TMEM176A) was first identified by screening tumor related antigens in hepatocellular carcinoma (HCC) [16, 17]. TMEM176A is located in human chromosome 7q36.1, a region which shows frequent loss of heterozygosity in human esophageal cancer [18–20]. In our recent study, loss of TMEM176A expression was shown to be a frequent event in human colorectal cancer by transcriptome analysis [Epigenetics 2017, in press]. The role and mechanism of TMEM176A in human esophageal carcinogenesis and development remain unclear. In this study, we investigated the epigenetic regulation and function of TMEM176A in esophageal cancer.

## RESULTS

### The expression of TMEM176A is regulated by promoter region methylation in human ESCC

**Figure 1:**
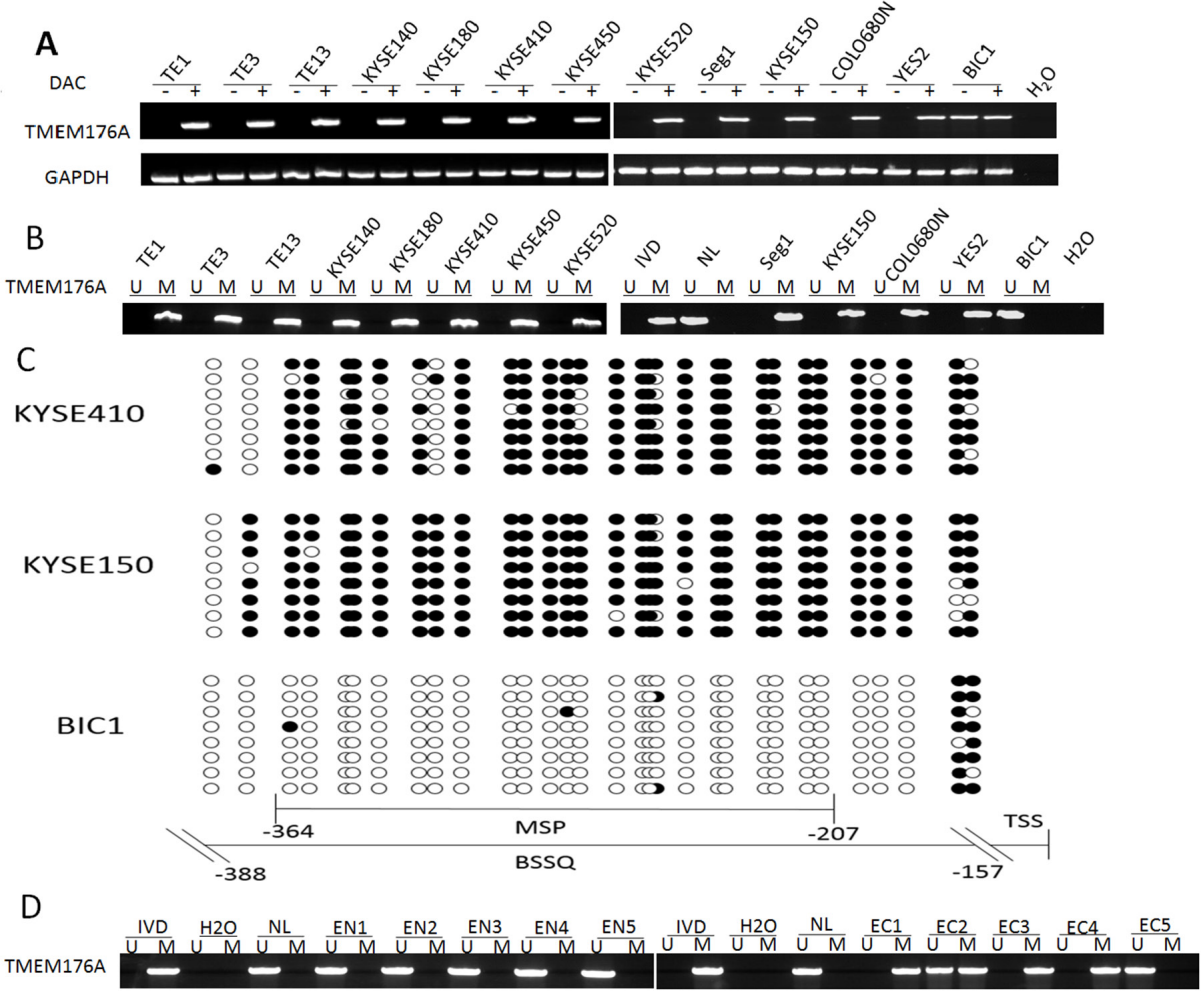
The expression and methylation status of TMEM176A in esophageal cancer **(A)** Semi-quantitative RT-PCR shows TMEM176A expression levels in esophageal cancer cell lines. TE1, TE3, TE13, KYSE140, KYSE180, KYSE410, KYSE450, KYSE520, Seg1, KYSE150, COLO680N, YES2 and BIC1 are esophageal cancer cell lines. DAC: 5-aza-2'-deoxycytidine; GAPDH: internal control of RT-PCR; H_2_O: double distilled water. (-): absence of DAC; (+): presence of DAC. **(B)** MSP results of TMEM176A in esophageal cancer cell lines. U: unmethylated alleles; M: methylated alleles; IVD: *in vitro* methylated DNA, serves as methylation control; NL: normal peripheral lymphocytes DNA, serves as unmethylation control; H_2_O: double distilled water. **(C)** BSSQ results of TMEM176A. NE: normal esophageal mucosa. Double-headed arrow: MSP PCR product spanned 157 bp in TMEM176A. Bisulfite sequencing focused on a 287 bp region of the CpG island (-388 bp to -157 bp) across the TMEM176A transcription start site. Filled circles: methylated CpG sites, open circles: unmethylated CpG sites. TSS: transcription start site. **(D)** Representative MSP results of TMEM176A in normal esophageal mucosa (EN1, EN2, EN3, EN4 and EN5) and primary esophageal cancer tissues (EC1, EC2, EC3, EC4 and EC5).

The expression of TMEM176A was detected by semi-quantitative RT-PCR in human esophageal cancer cell lines. As shown in Figure 1A, TMEM176A was highly expressed in BIC1 cells, and no expression of TMEM176A was detected in TE1, TE3, TE13, KYSE140, KYSE180, KYSE410, KYSE450, KYSE520, Segl, KYSE150, YES2 and COLO680N cells. TMEM176A promoter region methylation was examined by MSP. Complete methylation was found in TE1, TE3, TE13, KYSE140, KYSE180, KYSE410, KYSE450, KYSE520, Segl, KYSE150, YES2, and COLO680N cells, while unmethylation was detected in BIC1 cells (Figure 1B). These results demonstrated that loss of TMEM176A expression is related to promoter hypermethylation in esophageal cancer cells. To validate the efficiency of the MSP primers, bisulfite sequencing was employed. Dense methylation was observed in the promoter region of TMEM176A in KYSE150 and KYSE410 cells, and unmethylation was found in BIC1 cells (Figure 1C). To further investigate the regulation of TMEM176A, esophageal cancer cells were treated with 5-aza-2’-deoxycytidine (DAC), an inhibitor of DNA methyltransferases. The expression of TMEM176A was induced by DAC in TE1, TE3, TE13, KYSE140, KYSE180, KYSE410, KYSE450, KYSE520, Seg1, KYSE150, COLO680N and YES2 cells (Figure 1A). These results further suggest that the expression of TMEM176A is regulated by promoter region methylation.

### TMEM176A is frequently methylated in human ESCC and methylation of TMEM176A is an independent prognostic factor for 5-year overall survival (OS)

The methylation status of TMEM176A was detected by MSP in 267 cases of primary ESCC and 27 cases of esophageal mucosa from non-cancerous patients. TMEM176A was methylated in 66.7% (178/267) of esophageal cancer samples and no methylation (0/27) was found in non-cancerous esophageal mucosa (Figure 1D).

Methylation of TMEM176A was significantly associated with tumor cell differentiation (*p<0.01*, Table 1), no association was found between TMEM176A methylation and age, gender, lymphatic node metastasis, TNM stage, drinking history, family history, smoking history and tumor size (all *p* > 0.05, Table 1).

**Table 1:** The association of TMEM176A methylation and clinical factors in esophageal cancer

Clinical parameter	NO.	Methylation status		*P* value*
		Methylated	Unmethylated	
		n=178	n=89	
**Gender**				
Male	167	112	55	p=0.964
Female	100	66	34	
**Age**				
≥60	128	82	46	p=0.462
<60	139	96	43	
**Differentiation**				
Poorly	84	66	18	p=0.008**
Middle/high	183	112	71	
**Tumor stage**				
I/II	160	111	49	p=0.309
III/IV	107	67	40	
**Lymph node Metastasis**				
Positive	177	112	65	p=0.131
Negative	90	66	24	
**Drinking history**				
Yes	66	46	20	p=0.652
No	201	132	69	
**Family history**				
Yes	113	79	34	p=0.405
No	154	99	55	
**Smoking history**				
Yes	103	73	30	p=0.65
No	164	105	59	
**Tumor size**				
≥3cm	121	87	34	p=0.128
<3cm	146	91	55	

*p values are obtained from chi-square test and the Fisher’s exact test, significant difference, *p*< 0.05.

**Figure 2:**
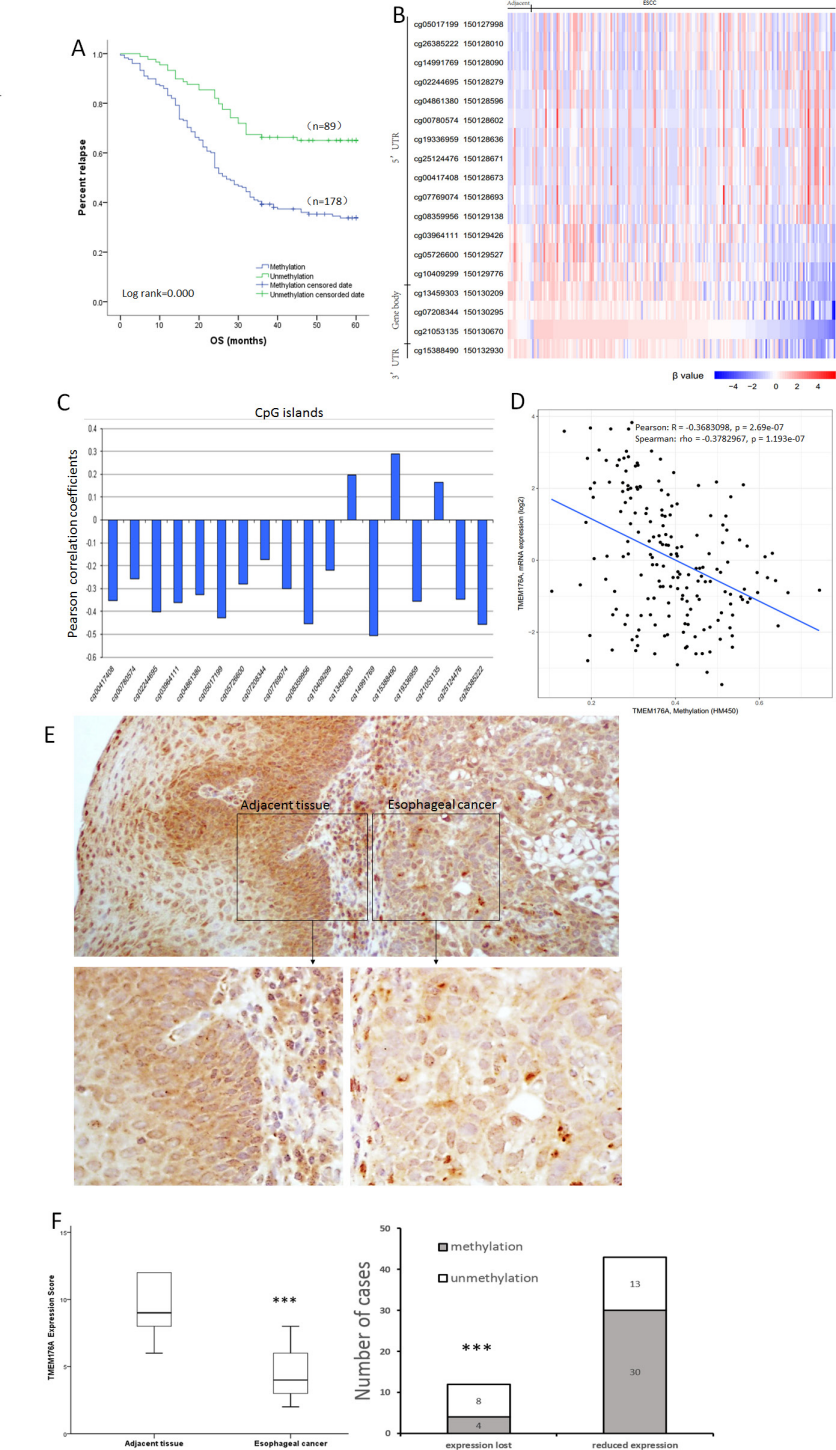
Methylation status and expression of TMEM176A in primary esophageal cancer samples **(A)** TMEM176A methylation is associated with poor 5-years OS. OS: Overall survival. **(B)** Heatmap of methylation of 18 CpG sites in the promoter region of TMEM176A gene. **(C)** Correlation of methylation of each CpG site and expression of TMEM176A. **(D)** Scatter-plot: The methylation status of 18 CpG sites in the promoter region is correlated to loss off/reduced TMEM176A expression in 184 cases of esophageal cancers (Pearson: R = -0.3683098, *p* = 0.000 Spearman: rho = -0.3782967, *p* = 0.000). **(E)** Representative IHC results showing TMEM176A expression in esophageal cancer and matched adjacent tissue samples (upper: ×100; lower: ×400). **(F)** TMEM176A expression scores are shown as box plots, horizontal lines represent the median score; the bottom and top of the boxes represent the 25^th^ and 75^th^ percentiles, respectively; vertical bars represent the range of data. The expression levels of TMEM176A were significantly different between adjacent tissue and esophageal cancer samples. ***p< 0.01.

The risk factor of OS was analyzed by Kaplan-Meier survival analysis. Under univariate analysis, TMEM176A methylation (hazard ratio= 2.25, *p<* 0.01) and tumor differentiation (hazard ratio= 1.841, *p<* 0.01) were risk factors for poor 5-years OS. Under multivariate analysis, the risk factors of poor OS were TMEM176A methylation (hazard ratio= 2.237, *p*<0.01, Table 2) and tumor differentiation (hazard ratio= 1.894, *p*<0.01, Table 2). In 178 cases of TMEM176A methylated patients, the mean time was 33.875 months and cumulative 5-years OS rate was 34.8%. In 89 cases of TMEM176A unmethylated patients, the mean time was 46.914 months and cumulative 5-years OS rate was 65.2%, log-tank= 0.000 (Figure 2A, Table 3). Cox proportional hazards model analysis indicated that TMEM176A methylation is an independent prognostic factor for poor 5-years OS (*p* < 0.05, Figure 2A, Table 3).

**Table 2:** Univariate and multivariate analysis of clinic pathologic factors for overall survival in 267 patients with esophageal cancer

Risk factors	OS					
	Univariate analysis			Multivariate analysis		
	HR	*P*^*a*^	95%CI	HR	*P*^*a*^	95%CI
**Age**	0.839	0.325	**0.592-1.189**			
**Gender**	0.797	0.198	**0.564-1.126**			
**TNM stage**	1.062	0.728	**0.758-1.486**			
**Tumor differentiation**	1.841	0.001^**^	**1.288-2.632**	1.894	0.000^**^	**1.345-2.65**
**Lymph node Metastasis**	0.845	0.358	**0.589-1.211**			
**TMEM176A methylation**	2.250	0.001^**^	**1.383-3.661**	2.237	0.000^**^	**1.496-3.345**
**Drinking history**	1.211	0.380	**0.789-1.859**			
**Family history**	1.233	0.218	**0.884-1.721**			
**Smoking history**	0.871	0.490	**0.590-1.288**			
**Tumor size**	0.967	0.840	**0.701-1.334**			

HR: hazard ratio. *p < 0.05. **p < 0.01.

**Table 3:** Means and medians for survival time

Methylation/Unmethylation	Cumulative survival(%)	Mean^a^				Median^a^			
		Estimate	Std. error	95% CI		Estimate	Std. error	95% CI	
				Lower bound	Upper bound			Lower bound	Upper bound
Methylation	62/178(34.8)	33.875	1.579	30.779	36.970	27.000	2.144	22.798	31.202
Unmethylation	58/89(65.2)	46.914	1.983	43.027	50.800				
Overall	120/267(44.9)	38.238	1.299	35.691	40.784	34.000	7.177	19.934	48.066

a. Estimation is limited to the largest survival time if it is censored.

As shown in Figure 2B, 2C & 2D, our results were supported by The Cancer Genome Atlas database (https://cancergenome.nih.gov/). Methylation of 18 CpG sites in the promoter region was associated to loss off/reduced expression of TMEM176A in 184 cases of esophageal cancers (Pearson: R= -0.3683098, *p=* 0.000, Spearman: rho= -0.3782967, *p=* 0.000).

The expression of TMEM176A was evaluated by immunohistochemistry (IHC) in 55 cases of available matched ESCC and adjacent tissue samples. TMEM176A staining was observed mainly in the cytoplasm and cell membrane of the esophageal cancer cells. TMEM176A was expressed in adjacent tissue samples and its expression was reduced in primary cancer samples (Figure 2E). Among the 43 cases in which TMEM176A expression was reduced, 30 cases were methylated. Reduced expression of TMEM176A was significantly associated with promoter region hypermethylation (Figure 2F, *p*<0.05). These results indicate that the expression of TMEM176A is regulated by promoter region methylation in primary esophageal cancer.

### TMEM176A suppresses esophageal cancer cell proliferation

**Figure 3:**
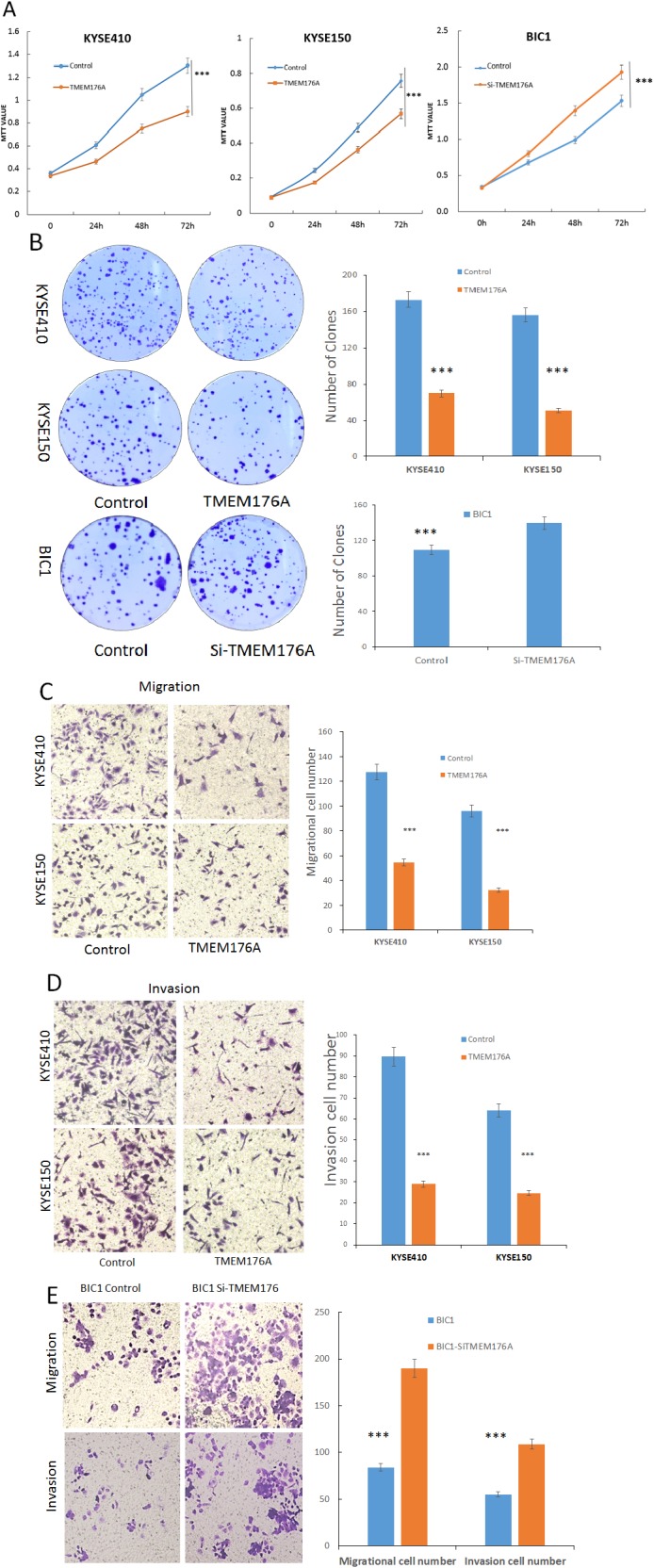
Representative results of MTT assay, colony formation, migration and invasion in esophageal cancer cells **(A)** Growth curves represent cell viability analyzed by the MTT assay in TMEM176A unexpressed and re-expressed KYSE410 and KYSE150 cells, as well as before and after siRNA knockdown of TMEM176A in BIC1 cells. The experiment was repeated for three times (***p<0.01). **(B)** Colony formation assays show colony number in TMEM176A unexpressed and re-expressed KYSE150 and KYSE410 cells. Each experiment was repeated for three times. The average number of tumor clones is represented by bar diagram. As well as before and after siRNA knockdown of TMEM176A significantly promotes colony formation in BIC1 cells (***p<0.01). **(C)** Cell migration in TMEM176A unexpressed and re-expressed KYSE150 and KYSE410 cells. The ratio is presented by bar diagram. Each experiment was repeated three times (***p<0.01). **(D)** Cell invasion in TMEM176A unexpressed and re-expressed KYSE150 and KYSE410 cells. The ratio is presented by bar diagram. Each experiment was repeated three times (***p<0.01). **(E)** Cell migration and cell invasion in before and after siRNA knockdown of TMEM176A and restoration of TMEM176A expression BIC1. The ratio is presented by bar diagram. Each experiment was repeated three times (***p<0.01).

To evaluate the effects of TMEM176A on cell viability, the MTT assay was employed. The OD values were 1.301 ± 0.089 vs. 0.9 ± 0.06 (*p*<0.01) and 0.758 ± 0.046 vs. 0.567 ± 0.019 (*p*<0.01) before and after restoration of TMEM176A expression in KYSE150 cells and KYSE410 cells. The effect of TMEM176A on cell growth was further validated by knocking down TMEM176A in BIC1 cells. The OD values were 1.585 ± 0.162 vs. 1.983 ± 0.055 (*p*<0.01) before and after knockdown TMEM176A in BIC1 cells (Figure 3A). These results demonstrate that TMEM176A suppresses esophageal cancer cell viability.

To evaluate the effects of TMEM176A on clonogenicity in esophageal cancer, we performed colony formation assays. The clone numbers were 173.7 ± 9.9 vs. 70.3 ± 5.7 in KYSE410 cells (*p*<0.01) and 155.7 ± 6.8 vs. 50.3 ± 3.7 in KYSE150 cells (*p*<0.01) before and after restoration of TMEM176A expression, respectively (Figure 3B). To further validate the effect of TMEM176A on clonogenicity, siRNA knockdown technique was employed. The clone number was 108.3 ± 11.4 vs. 139.0 ± 6.0 (*p*<0.05) before and after knockdown of TMEM176A in BIC1 cells (Figure 3B). These results suggest that TMEM176A inhibits cell proliferation in esophageal cancer.

### TMEM176A suppresses cell migration and invasion in esophageal cancer cells

To evaluate the effects of TMEM176A on cell migration and invasion, transwell assay was employed. The number of migratory cells was 125.7 ± 5.2 vs. 51.0 ± 5.6 in KYSE410 cells (*p*<0.01) and 94.7 ± 3.6 vs. 37.3 ± 4.7 in KYSE150 cells (*p*<0.01) before and after restoration of TMEM176A expression. The number of migratory cells was 78.7 ± 5.2 vs. 193.0 ± 5.3 before and after knockdown of TMEM176A in BIC1 cells (*p*<0.01, Figure 3E). The number of migratory cells was significantly reduced after restoration of TMEM176A expression in KYSE410 and KYSE150 cells (Figure 3C). These results suggested that TMEM176A suppresses esophageal cancer cell migration. The number of invasive cells was 90.0 ± 14.1 vs. 25.3 ± 1.1 in KYSE410 cells (*p*<0.01) and 55.0 ± 7.1 vs. 29.7 ± 3.2 in KYSE150 cells (*p*<0.01) before and after restoration of TMEM176A expression. The number of invasive cells was significantly reduced after restoration of TMEM176A expression in KYSE410 and KYSE150 cells (Figure 3D). The number of invasive cells was 55.3 ± 6.0 vs. 105.3 ± 4.5 before and after knockdown of TMEM176A in BIC1 cells (*p*<0.01, Figure 3E). These results suggest that TMEM176A suppresses esophageal cancer cell invasion.

### TMEM176A induces apoptosis in esophageal cancer cells

**Figure 4:**
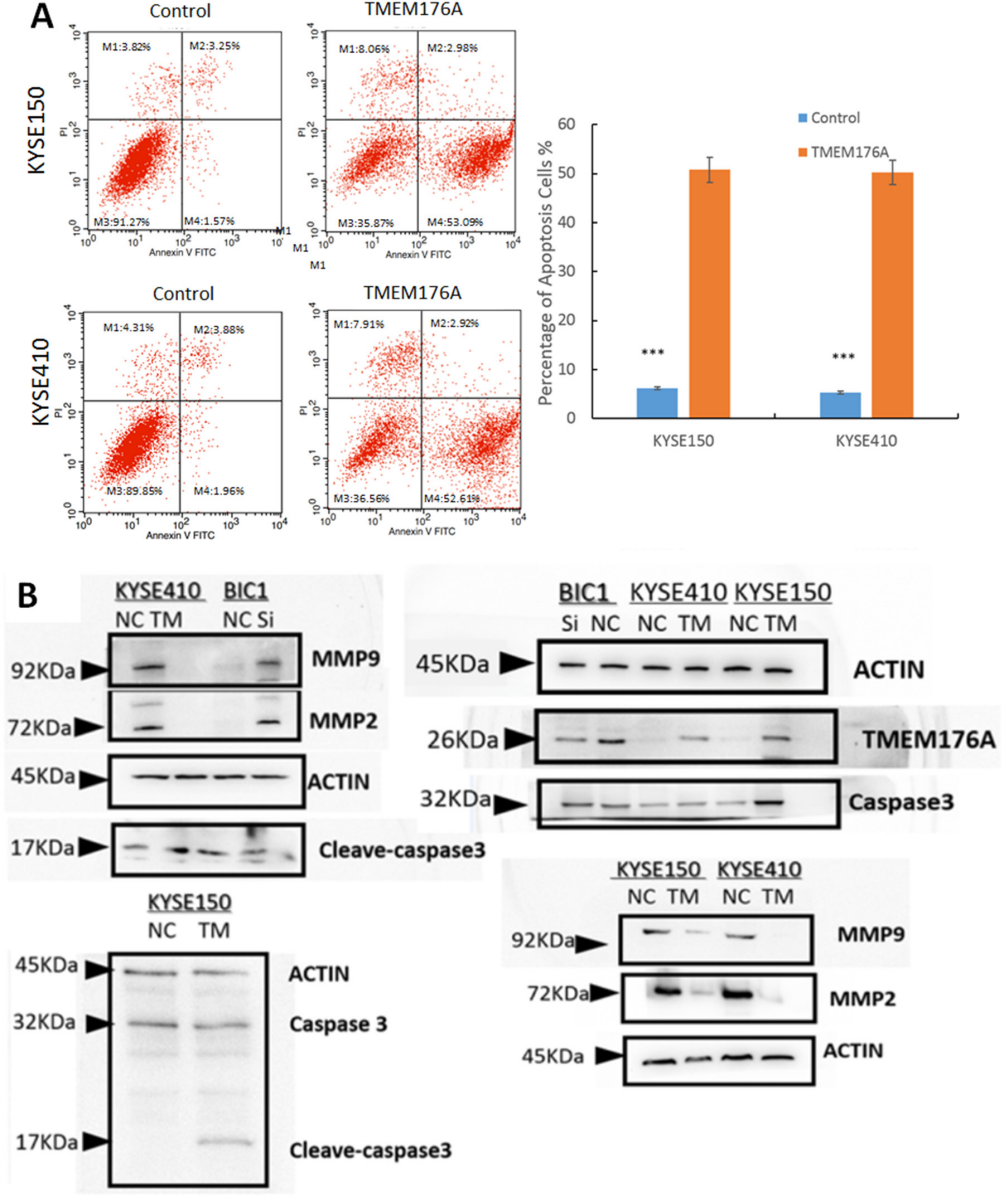
Representative results of cell apoptosis and western blot in esophageal cancer cells **(A)** DOX induces apoptosis in TMEM176A unexpressed and re-expressed KYSE150 and KYSE410 cells were evaluated in stably transfected cells after 24h by flow cytometry analysis. The ratio is presented by bar diagram. ***p<0.01. **(B)** The expression levels of TMEM176A, MMP-2, MMP-9, Caspase 3 and cleaved-caspase 3 were detected by western blot in TMEM176A unexpressed and re-expressed KYSE150 and KYSE410 cells. Knockdown of TMEM176A by siRNA was performed to validate the results in TMEM176A highly expressed BIC1 cells.

To evaluate the role of TMEM176A in cell apoptosis, flow cytometry assay was used. The percentage of apoptotic cells was 5.2 ± 1.1% *vs.* 48.8 ± 6.1% in TMEM176A unexpressed and re-expressed KYSE410 cells. The percentage of apoptotic cells increased significantly after restoration of TMEM176A expression in KYSE410 cells (*p*<0.01, Figure 4A). In KYSE150 cells, the percentage of apoptotic was 4.8 ± 1.0% vs. 51.8 ± 3.1% before and after restoration of TMEM176A expression. The percentage of apoptotic cells increased significantly after re-expression of TMEM176A in KYSE150 cells (*p*<0.01, Figure 4A). As shown in Figure 4B, the levels of cleaved-caspase 3 increased after restoration of TMEM176A expression in KYSE410 and KYSE150 cells, and the levels of cleaved-caspase 3 decreased after knockdown of TMEM176A in BIC1 cells. These results suggest that TMEM176A induced cell apoptosis in esophageal cancer. MMP2 and MMP9 expression levels were detected by western blot to validate the effects of TMEM176A on cell migration and invasion. The levels of MMP2 and MMP9 expression were reduced after restoration of TMEM176A expression in KYSE410 and KYSE150 cells. To further validate the role of TMEM176A in cell invasion and migration, siRNA knockdown technique was employed. MMP2 and MMP9 expression levels were increased after knockdown of TMEM176A in BIC1 cells (Figure 4B).

### TMEM176A suppresses tumor growth in esophageal cancer cell xenograft mice

**Figure 5:**
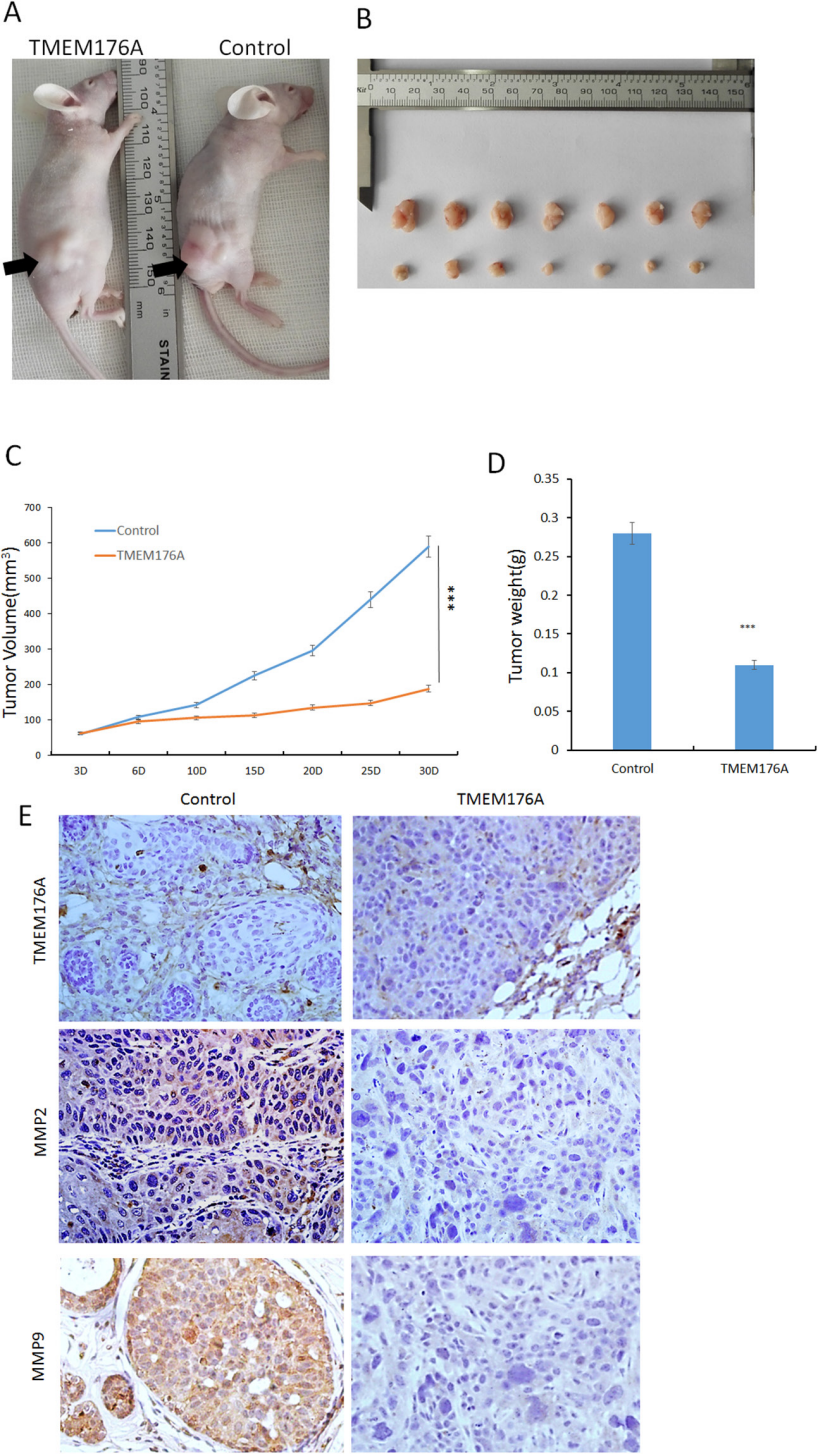
TMEM176A inhibits tumor growth in esophageal cancer cell xenograft mice **(A)** Representative nude mice burdened with TMEM176A unexpressed and re-expressed KYSE410 cells, the tumor location is shown by black arrowhead. **(B)** Results of TMEM176A restoration of TMEM176A unexpressed and re-expressed KYSE410 cells, xenografts in mice – Bottom: TMEM176A restoration of TMEM176A expression cells group; Top: control group. **(C)** The volumes of xenograft tumors in TMEM176A unexpressed and re-expressed KYSE410 cells after inoculation for four weeks. ***p<0.01. **(D)** Tumor weights from nude mice at the 30th day after inoculation with TMEM176A unexpressed and re-expressed KYSE410 cells. ***p<0.01. **(E)** IHC staining reveals the expression levels of TMEM176A, MMP2, MMP9 in TMEM176A unexpressed and re-expressed KYSE410 cell xenografts (200×).

To further explore the impacts of TMEM176A on esophageal cancer, a xenograft mouse model was established (Figure 5A). The tumor volumes were 257.77 ± 164.65 mm^3^ and 129.72 ± 45.22 mm^3^ in TMEM176A unexpressed and re-expressed KYSE410 cell xenografts, respectively. The tumor volume was significantly smaller in TMEM176A re-expression KYSE410 cell xenografts compared to TMEM176A unexpressed KYSE410 cell xenografts (*P*<0.05, Figure 5B & 5C). The tumor weights were 0.27 ± 0.07g and 0.12 ± 0.03g in TMEM176A unexpressed and re-expressed KYSE410 cell xenografts, respectively. The tumor weight was significantly different (*P*<0.05, Figure 5B & 5D), suggesting that TMEM176A suppresses esophageal cancer cell tumor growth *in vivo*. To further validate the effect of TMEM176A on MMP2 and MMP9 *in vivo*, the expression of MMP2 and MMP9 were examined by IHC staining in xenograft tumors. TMEM176A was expressed in TMEM176A re-expressed KYSE410 cell xenografts, and it was unexpressed in KYSE410 parental cell xenografts (Figure 5E, upper panels). The expression levels of MMP2 and MMP9 were decreased in TMEM176A re-expressed KYSE410 cell xenografts compared to parental cells (Figure 5E, middle & low panels). The above results suggest that TMEM176A suppresses esophageal cancer cell growth and invasion *in vivo*.

## DISCUSSION

TMEM176A was reported in a few studies [16]. While, the functional study was very limited and the research work was mainly focused on development and the immune system. Tmem176a is highly expressed in RORγt+ (Retinoid-related orphan receptor gamma t) lymphocytes in mice [21]. Tmem176a induces the expression of co-stimulatory molecules, such as Cluster of Differentiation (CD)-80, CD86, and CD40 and is involved in the maintenance of the immature state of dendritic cells in mice [22-23]. By analyzing amino acid sequence, a great similarity (28.5% identity) was found with TMEM176B in human. Among TMEM176A, TMEM176B and 12 known MS4A family members, the four predicted transmembrane regions (TM1-4) are similar in size and structure in human, rat and mouse [24]. It was reported that Tmem176a and its homologue, Tmem176b, are located within the same genomic locus in opposite directions and they are tightly co-regulated in various tissues in mice [25]. Drujont et al. found that the expression of Tmem176a was increased in Tmem176b −/− cells compared to wildtype cells in Th17 cells in mice [21], while no association was found between TMEM176A and TMEM176B expression in human esophageal cancer cells in our study.

Although human TMEM176A was identified in 2002, no direct evidence has been established indicating that TMEM176A is a cancer related protein. Evidence on the functional relevance of TMEM176A in cancer development is also lacking. Loss of/reduced expression of TMEM176A was found in colorectal cancer by our transcriptome study (*Epigenetics2017*, in press). In this study, we found that the expression of TMEM176A is frequently lost in esophageal cancer cells, and the expression of TMEM176A is regulated by promoter region methylation. TMEM176A was methylated in 66.7% human primary esophageal cancer. Thus, TMEM176A methylation may serve as an esophageal cancer detection marker. Methylation of TMEM176A was significantly associated with tumor differentiation and poor 5-years OS. Cox proportional hazards model analysis suggest that TMEM176A methylation is an independent prognostic factor for poor 5-years OS. Thus, TMEM176A methylation may serve as a prognostic marker in ESCC. The levels of TMEM176A expression were significantly lower in esophageal cancer tissue samples compared to matched adjacent tissue samples. The reduced expression of TMEM176A was significantly associated with promoter region hypermethylation. These results suggest that the expression of TMEM176A is silenced by promoter region hypermethylation in primary human esophageal cancer. These results were supported by the TCGA database analysis. Further experiments indicated that TMEM176A induces apoptosis and inhibits esophageal cancer cell proliferation, invasion and migration. TMEM176A suppresses esophageal cancer cell tumor growth in xenograft mice. Taken together, our results demonstrate that TMEM176A methylation is frequently methylated in human ESCC and TMEM176A expression is regulated by promoter region methylation. TMEM176A methylation may serve as a diagnostic and prognostic marker. TMEM176A is a potential tumor suppressor in human ESCC.

## MATERIALS AND METHODS

### Human tissue samples and cell lines

Thirteen esophageal cancer cell lines (TE1, TE3, TE13, KYSE140, KYSE180, KYSE410, KYSE450, KYSE520, Segl, KYSE150, YES2, COLO680N and BIC1) were included in this study. All esophageal cancer cell lines were previously established from primary esophageal cancer and maintained in 90% RPMI media 1640 (Invitrogen, CA, USA) supplemented with 10% fetal bovine serum. Cells were passaged 1:3 when total confluence was reached in a 75cm^2^ culture flask (NEST Biotechnology, Jiangsu, China). All cell lines were cultured in an atmosphere of 5% carbon dioxide at 37°C.

A total of 267 cases of primary esophageal cancer samples and 27 cases of normal esophageal mucosa from patients without cancer were collected from the Chinese PLA General Hospital in Beijing. Among the 267 patients, 167 cases were male and 100 cases were female. The median age was 61.7 years old (range 37–85 years old). All tumors were classified according to the TNM staging system (AJCC2010), including stage I (n=36), II (n=124), III (n=103), IV (n=4). Snap-frozen fresh tissue samples were collected by surgical resection and stored at -80°C. All samples were collected under the guidelines approved by the institutional review board at the Chinese PLA General Hospital.

### DAC treatment

Esophageal cancer cell lines were split to a low density (30% confluence) 12 hours before treatment. Cells were treated with DAC (5-aza-2’-deoxycytidine, Sigma, St. Louis, MO, USA) at a concentration of 2 μM which was exchanged every 24 h for a total 96 h treatment. At the end of the treatment course, RNA was isolated as described below.

### RNA isolation and semi-quantitative RT-PCR

Total RNA was isolated by Trizol reagent (Life Technology, MD, USA). Agarose gel electrophoresis and spectrophotometric analysis were used to check RNA quality and quantity. Total RNA (5μg) was used to synthesize first strand cDNA according to the manufacturer’s instructions (Invitrogen, Carlsbad, CA). The reaction mixture was diluted to 100μl with water, and 2.5μl of diluted cDNA mixture was added to each 25μl PCR reaction. The TMEM176A PCR primer sequences were as follows: 5’-GGGAACAGCCGACAGTGAT-3’ (F) and 5’-GCCAGCGTTAGCAGAGTCCT-3’ (R).

Products were amplified for 35 cycles. GAPDH was amplified for 25 cycles as an internal control. The primer sequences for GAPDH were as follows: 5’-GAC CAC AGT CCA TGC CAT CAC-3’ (F), and 5’-GTC CAC CAC CCT GTT GCT GTA-3’ (R). The amplified PCR products were examined by 1.5% agarose gels.

### Bisulfite modification, methylation-specific PCR and bisulfite sequencing

Genomic DNA was extracted by the proteinase K method. The bisulfite modification assay was performed as previously described [26]. Methylation specific PCR (MSP) primers were designed according to genomic sequences around the transcription start sites (TSS) and synthesized (BGI, Beijing, China) to detect unmethylated (U) and methylated (M) alleles. MSP primer sequences were as follows: 5’-GTTTCGTTTAGGTTGCGCGGTTTTTC-3’(MF) and 5’-CCAAAACCGACGTACAAATATACGCG-3’(MR); 5’-TGGTTTTGTTTAGGTTGTGTGGTTTTTT-3’(UF) and 5’-CAACCAAAACCAACATACAAATATACACA-3’(UR). The expected sizes of unmethylated and methylated products were 154bp and 159bp, respectively. Bisulfite-treated DNA was also amplified using bisulfite sequencing (BSSQ) primers that included the MSP region. The sequencing primers were as follows: 5’-AGAATGTTCCCAACCAAAGGGA-3’(F) and 5’-TGGGGAAGGGGTGTAAGGAAT-3’(R). Bisulfite sequencing was performed as previously described [27].

### Immunohistochemistry

Immunohistochemistry (IHC) was performed in primary esophageal cancer and paired adjacent tissue samples. TMEM176A antibody was diluted to 1/150 dilution (Anti-TMEM176A, Abcam, USA). The staining intensity and extent of the stained area were scored using the German semi-quantitative scoring system. The staining intensity of TMEM176A expression was quantified as follows: no staining = 0; weak staining = 1; moderate staining = 2; strong staining = 3. The extent of TMEM176A expression was quantified as follows: 0% = 0, 1–24% = 1, 25–49% = 2, 50–74% = 3, 75–100% = 4 [28, 29]. The final immune-reactive score (0 to 12) was determined by multiplying the intensity score to the extent of stained cells score.

### Construction of Lentiviral TMEM176A expression vectors and selection of stable expression cells

The human full length TMEM176A cDNA (GenBank accession number NM_018487.2) was cloned into the pLenti6-GFP vector according to our previous report [30]. Primers were as follows: 5’-CTTAGGATCCGCCACCATGGGAACAGCCGAC-3’(F) and 5’-ACTTAGTCGACCTAGATTCCACTCACTTCC-3’(R). The HEK-293T cell line was maintained in 90% DMEM (Invitrogen, Carlsbad, CA, USA) supplemented with 10% fetal bovine serum. TMEM176A expressing Lentiviral vector was transfected into HEK-293T cells (5×10^6^ per 100mm dish) using Lipofectamine 3000 Reagent (Invitrogen, CA, USA) at a ratio of 1:3 (DNA mass: Lipo mass). Viral supernatant was collected and filtered after 48 hours. KYSE410 and KYSE150 cell lines were then infected with viral supernatant. Cells stably expressing TMEM176A were selected with Blasticidin (Life Technologies, Gaithersburg, MD, USA) at concentrations of 0.5μg/ml (KYSE410) and 0.4μg/ml (KYSE150) for 2 weeks.

### Cell viability assay

Cells were plated into 96-well plates at a density of 3×10^3^ cells/well, and the cell viability was measured by the MTT assay at 0, 24, 48 and 72h (KeyGEN Biotech, Nanjing, China). Absorbance was measured on a microplate reader (Thermo Multiskan MK3, MA, USA) at a wavelength of 490 nm. The results were plotted as means ± SD.

### Colony formation assay

TMEM176A stably expressed and controled KYSE410 and KYSE150 cell lines were seeded in 6-well plates at a density of 1000 cells per well. Growth medium, which included blasticidin at 0.5μg/ml (KYSE410) or 0.4μg/ml (KYSE150), was exchanged every 24 hours. After 14 days, cells were fixed with 75% ethanol for 30min and stained with 0.2% crystal violet. The number of clones was then counted. Each experiment was repeated three times.

### Flow cytometry

TMEM176A stably restoration of TMEM176A expressioned and controled KYSE410 and KYSE150 cells were serum starved for 12 hours for synchronization, and cells were re-stimulated with 10% FBS add DOX 10 ug for 24 hours. Cells were fixed with 70% ethanol and treated using the Cell Cycle Detection Kit (KeyGen Biotech, Nanjing, China). Cells were then analyzed using a FACS Caliber flow cytometer (BD Biosciences, Mansfield, CA). Cell phase distributions were analyzed using the Modfit software (Verity Software House, ME, USA).

### Transwell assay

Migration: 1×10^5^ TMEM176A controled and restoration of TMEM176A expressioned KYSE410 and KYSE150 cells were suspended in 200μl serum-free RPMI 1640 media and added to the upper chamber of an 8.0μm pore size transwell apparatus (COSTAR Transwell, Corning Incorporated, MA, USA). Cells that migrated to the lower surface of the membrane were stained with crystal violet and counted in three independent high-power fields (×200) after incubating for 17 hours.

Invasion: the top chamber was coated with a layer of extracellular matrix. Cells (2×10^5^) were seeded into the upper chamber of a transwell apparatus coated with Matrigel (BD Biosciences, SanJose, CA) and incubated for 36 hours. Cells that invaded to the lower membrane surface were stained with crystal violet and counted in three independent high-power fields (×200).

### SiRNA knockdown technique

Selected siRNAs targeting TMEM176A and RNAi negative control duplex were used in this study. The sequences were as follows: siRNA duplex (sense: 5’-CUG UAC UGC UGG AGA AUG UTT-3’; antisense: 5’-ACA UUC UCC AGC AGU ACA GTT-3’); RNAi negative control duplex (sense: 5’-UUC UCC GAA CGU GUC ACG UTT-3’; antisense: 5’-ACG UGA CAC GUU CGG AGA ATT-3’). RNAi oligonucleotide or RNAi negative control duplex (Gene Pharma Co. Shanghai, China) were transfected into TMEM176A highly expressed BIC1cells.

### Protein preparation and western blot

Protein samples from controled and stably restoration of TMEM176A expressioned KYSE410 and KYSE150 cells were collected and western blots were performed as described previously [28]. Antibodies were diluted according to the manufacturer’s instructions. Primary antibodies included TMEM176A, Caspase 3, Cleaved-caspase 3, MMP2, MMP9 (Bioworld Tech, MN, USA), and β-actin (Beyotime Biotech, Jiangsu, China).

### The effects of TMEM176A on KYSE410 cell xenograft

Stably transfected KYSE410 cell line with pLenti6-vector or pLenti6-TMEM176A/Flag vector (4×10^6^ cells in 0.15ml phosphate-buffered saline) were injected subcutaneously into the dorsal left side of 4–week-old male BABL/c nude mice (n=7). Tumor volumes were measured every 3-5 days for 27 days starting 3 days after implantation. Tumor volume was calculated according to the formula: V=L×W^2^/2, where V represents volume (mm^3^), L represents biggest diameter (mm), and W represents smallest diameter (mm). All procedures were approved by the Animal Ethics Committee of the Chinese PLA General Hospital.

### Clinical factors and statistical analysis

The association between TMEM176A methylation and clinical factors was analyzed, including age, gender, TNM stage, lymph node metastasis, habitual drinking history (alcohol > 150 g/d, or beer >4 bottles/d, continue > 3 years), family history (Immediate family members suffer from esophageal cancer), habitual smoking history (Smoking cigarettes > 10/d, continue > 3 years), tumor size and tumor differentiation.

SPSS 22.0 software (IBM, NY, USA) was applied using χ^2^ test for independent dichotomous variables. All data were presented as means ± standard deviation (SD) of at least three independent experiments and analyzed using the student’s t test. Results were reported to be statistically significant at *p*<0.05(*), *p*<0.01(**). Survival rates were calculated by the Kaplan–Meier method, and differences in survival curves were evaluated using the log-rank test. Cox proportional hazards models were fit to determine independent associations of TMEM176A methylation with 5-year OS outcomes. Two-sided tests were used to determine significance, and *p*< 0.05 was considered statistically significant. The expression of TMEM176A and the methylation status in The Cancer Genome Atlas database were analyzed by the T test in ESCC.

## Abbreviations

DAC: 5-aza-2’-deoxycytidine
BSSQ: bisulfite sequencing
GAPDH: glyceraldehyde-3-phosphate dehydrogenase
ESCC: esophageal squamous cell carcinoma
IHC: immunohistochemistry
IVD: *in vitro* methylated DNA
ECM gel: extracellular matrix gel
MMP: matrix metalloproteinase
MSP: methylation specific polymerase chain reaction
TMEM: Transmembrane protein
NL: normal lymphocyte DNA
RT-PCR: reverse-transcription polymerase chain reaction
TGFα: transforming growth factor α
TSS: transcription start sites
